# Changes in Physician Emigration and Density After the 2010 WHO Global Code of Practice

**DOI:** 10.1001/jamahealthforum.2025.6718

**Published:** 2026-02-06

**Authors:** Tarun Ramesh, Joia S. Mukherjee, Fang Zhang, Anupam B. Jena, Hao Yu

**Affiliations:** 1 Department of Medicine, Massachusetts General Hospital, Boston; 2 Department of Population Medicine, Harvard Medical School and Harvard Pilgrim Health Care Institute, Harvard University, Boston, Massachusetts; 3 Department of Medicine, Brigham and Women’s Hospital, Boston, Massachusetts; 4 Department of Global Health and Social Medicine, Harvard Medical School, Harvard University, Boston, Massachusetts; 5 Department of Health Care Policy, Harvard Medical School, Harvard University, Boston, Massachusetts; 6 National Bureau of Economic Research, Cambridge, Massachusetts

## Abstract

**Importance:**

Physician emigration from low- and middle-income countries to high-income countries is a major driver of inequitable distribution of health care and health outcomes across the world. World Health Organization (WHO) signatory countries unanimously signed the voluntary Global Code of Practice on the International Recruitment of Health Personnel (WHO Code) in 2010. The goal was to reduce health care workforce emigration by discouraging active recruitment of physicians from WHO-designated shortage countries and by promoting investment in the physician workforce in those countries. This study adds to the literature by providing evidence about whether the goal has been achieved 10 years after worldwide implementation of the WHO Code.

**Objective:**

To evaluate whether the WHO Code was associated with changes in physician emigration and physician density in WHO-designated shortage countries after 2010.

**Design, Setting, and Participants:**

A difference-in-differences design was used to examine trends in physician supply before and after 2010. The data (from 2000 through 2021) were collected by the Organization for Economic Co-operation and Development (OECD) and were used to examine physician outflow from 56 WHO-designated shortage countries vs 116 nonshortage countries. The data analysis took place October 2024 to September 2025.

**Exposures:**

Worldwide adoption of the 2010 WHO Code.

**Main Outcomes and Measures:**

The primary outcome was annual physician migration to OECD countries and the secondary outcome was physician density by country and year (per 1000 population using World Bank data) in the origin countries.

**Results:**

A total of 135 888 physicians emigrated from WHO-designated shortage countries during 2000 to 2021 and 516 030 physicians emigrated from nonshortage countries. Compared with nonshortage countries, there was a decrease in physician outflow by 47.03 physicians (95% CI, −92.29 to −1.76 physicians) per country per year after 2010 in WHO-designated shortage countries and the WHO Code was associated with a reduction of nearly 30% in the average annual outflow of physicians from these countries. However, there was a slight decrease in physician density in the WHO-designated shortage countries after 2010 (−0.22 [95% CI, −0.33 to −0.11] physicians per 1000 population) compared with the nonshortage countries.

**Conclusions and Relevance:**

This study found that voluntary implementation of the WHO Code was associated with lower physician outflow from WHO-designated shortage countries without improvement in physician density in those countries.

## Key Points

**Question:**

Was the 2010 World Health Organization (WHO) Global Code of Practice on the International Recruitment of Health Personnel (WHO Code) associated with changes in physician migration and physician density in shortage countries?

**Findings:**

In this difference-in-differences analysis of 56 WHO-designated shortage countries (135 888 physicians) and 116 nonshortage countries (516 030 physicians), the WHO Code was associated with a reduction in annual physician emigration by nearly 30% during the first 5 years. However, physician density in shortage countries declined relative to nonshortage countries during the study period.

**Meaning:**

Although the WHO Code was associated with reductions in physician outflow from shortage countries, broader investments are needed to strengthen physician supply and health systems in those countries.

## Teaser

This difference-in-differences study evaluates whether the 2010 World Health Organization (WHO) Global Code of Practice on the International Recruitment of Health Personnel was associated with changes in physician emigration and physician density in WHO-designated shortage countries compared with nonshortage countries.

## Introduction

Physician shortage is a persistent global problem and remains an important barrier for improvements in health outcomes across the world.^1^ The World Health Organization (WHO) estimates a shortfall of approximately 10 million health care workers by 2030, with critical workforce shortages disproportionately affecting low- and middle-income countries (LMICs).^2^ Physician migration from LMICs to high-income countries (HICs) is a major driver of inequitable distribution of physicians across the world.

Most physicians in LMICs are trained with minimal charge at public institutions to serve their own country; however, many of these physicians immigrate to HICs for higher salaries, better working conditions, and improved career opportunities, contributing to physician emigration. To address these challenges, WHO signatory countries unanimously signed the voluntary Global Code of Practice on the International Recruitment of Health Personnel (WHO Code) in 2010, which set out 2 main objectives. First, the signatory countries committed to pursue more ethical international recruitment of health care workers, including reducing reliance on physicians from the WHO-designated shortage countries, while acknowledging freedom of migration. Second, the signatory countries committed to work together to increase the physician workforce in the WHO-designated shortage countries.^3^

Signing the WHO Code represented the first time that the international community agreed to take actions to reduce global health care workforce emigration and support physician workforce development in the WHO-designated shortage countries. However, there has been mixed evidence about the implementation of the WHO Code.^4,5,6^ Previous studies have highlighted that the voluntary implementation of the WHO Code was unlikely to shift destination country behavior, such as recruiting physicians from sub-Saharan African countries.^4^ A 2013 study found that among 56 surveyed countries, only 37 had taken steps to implement the first objective of the WHO Code (ie, reducing reliance on physicians from LMICs), whereas a 2016 study found ongoing barriers to the implementation in destination countries including Australia and the US.^7,8^ Prior studies also reported on the progress of implementing the WHO Code in several countries including Germany and Norway, which ratified key components to formally cease recruitment of physicians from WHO-designated shortage countries.^9,10^ In addition, bilateral agreements (including through academic medical centers in Rwanda and the US) have focused on training physicians who would gain international exposure and return to their origin country.^11^ Despite the existing research, no studies to date have assessed whether implementation of the WHO Code has achieved the intended objectives of reducing physician immigration from WHO-designated shortage countries and increasing physician supply in those countries.

The WHO Code remains relevant given the ongoing physician shortages and need for building capacity, especially for a robust physician workforce in LMICs that was brought into focus by the COVID-19 pandemic.^12^ In addition, the broader effectiveness of voluntary WHO policies has been called into question after the decision by the US to withdraw from the WHO, upending decades of global health governance and multilateralism.^13^ This continued relevance requires empirical study of WHO policies.

Although previous studies have identified progress in implementing the WHO Code, there has been a lack of empirical evidence about the effects of its implementation on physician recruitment by HICs from WHO-designated shortage countries and physician workforce growth in those countries. We evaluated whether the 2010 WHO Code was associated with reduced physician migration from WHO-designated shortage countries or changes in physician density (per 1000 population) in those countries from 2000 to 2021. A difference-in-differences study design was used to compare physician outflow and physician density in WHO-designated shortage countries and nonshortage countries (eLists in Supplement 1) before and after implementation of the WHO Code.

## Methods

### Data Sources and Study Outcomes

We used annual data from 2000 through 2021 collected by the Organization for Economic Co-operation and Development (OECD), a group that includes most of the world’s HICs. The OECD collects information about physicians immigrating to its member countries including information about physician origin countries.^14^ As the largest publicly available database about health care workforce migration, OECD data have previously been used to identify trends in internationally trained health care worker inflow to OECD countries.^15,16^ The primary outcome measure was the annual number of physicians that emigrated from WHO-designated shortage countries to OECD countries. In addition, using World Bank data, we constructed a secondary outcome measure of physician density (per 1000 population) by country and year.^17^

We followed the Strengthening the Reporting of Observational Studies in Epidemiology (STROBE) guideline. The data were publicly available without personal identifiers and the study was determined as not human subjects research by the Harvard Pilgrim Health Care Institute institutional review board.

### Statistical Analysis

We used a difference-in-differences study design to examine changes in physician supply after 2010.^18,19^ We defined the intervention period as a binary variable starting in 2010, the year when the WHO Code was signed. Of the 57 countries identified by the WHO as health care workforce shortage countries facing challenges with physician emigration and retention (eTables 1-6 in Supplement 1),^20^ 56 were included in the current analysis with complete data for the entire study period between 2000 and 2021 and were compared with 116 nonshortage countries.

We first conducted descriptive analyses by (1) comparing trends in annual physician outflow between shortage (treatment) and nonshortage (control) countries, (2) mapping shortage countries to identify top countries with physician emigration over the study period, and (3) mapping OECD countries to identify destination countries with the highest proportion of physicians from shortage countries (among the total number of physician immigrants to the destination country).

We then applied a difference-in-differences approach to compare changes in the study outcomes between shortage (treatment) and nonshortage (control) countries before and after the WHO Code in 2010. We estimated linear models with the following covariates from World Bank data to account for health system and population-level factors: male and female mortality per 1000, gross national income per capita, foreign direct investment, male and female unemployment rates, health expenditure per capita, out-of-pocket health care expenses per capita, population, and refugee population.^17^ The models also included fixed effects for country and year to account for time-invariant unobserved confounders. After the models were created to estimate and compare the period before and after 2010, we specified models to identify differential associations across 2 periods (2010-2014 and 2015-2021) and any interaction of the treatment variable with the 2-period variable. We confirmed the parallel trends assumption that was required for the difference-in-differences approach through placebo tests (eTable 7 in Supplement 1).

We conducted 5 sensitivity analyses. First, a sensitivity analysis was conducted that restricted the study period to before 2020. The restriction was due to the 2020 outbreak of the COVID-19 pandemic and the 2020 initiative to update the list of countries with health workforce shortages (known as the WHO Health Workforce Support and Safeguards List), which is updated every 3 years in response to changes in the health needs of populations (additional details appear in the Discussion section). Second, a sensitivity analysis was conducted that included a restricted control group in which only LMICs could be assigned. Third, a sensitivity analysis was conducted that used different cutoffs to define 2 periods (2010-2014 and 2015-2021) after implementation of the WHO Code. Fourth, a sensitivity analysis was conducted that used 2010 as the washout period because the WHO Code was unanimously signed in May 2010 and data for 2010 were split between the before WHO Code and the after period. Fifth, a matched difference-in-differences analysis was conducted by using nearest neighbor matching on all the covariates. Through this matching process, each shortage (treatment) country was able to match up to 3 nonshortage (control) countries and each treatment country was matched on the year of intervention (ie, 2010) with replacement. We identified 54 shortage countries and 111 nonshortage countries for the matched analysis.

All tests were 2 sided with an α level of .05 and were conducted using Stata version 18.0 (StataCorp). The data analysis took place October 2024 to September 2025.

## Results

**Figure 1.**
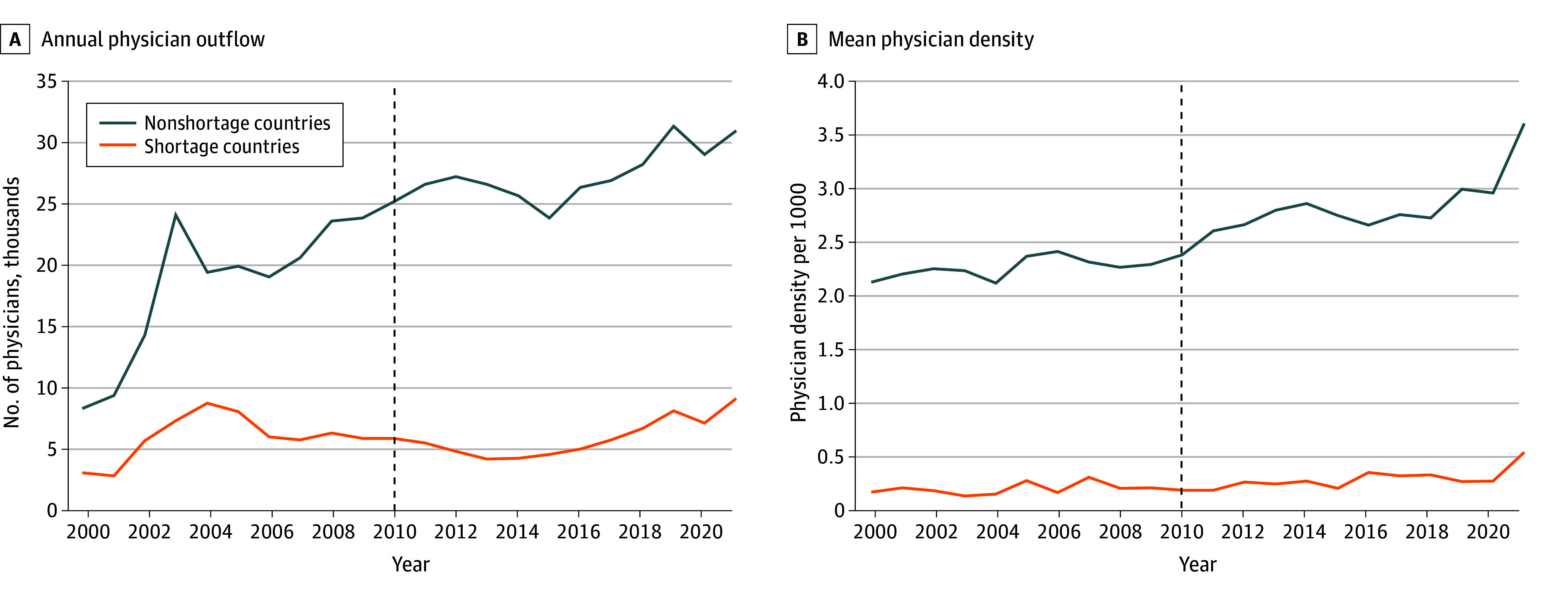
Annual Physician Outflow and Mean Physician Density in World Health Organization (WHO)–Designated Shortage and Nonshortage Countries A, Total physician outflow from nonshortage and shortage countries from 2000 to 2021. The dashed line represents the year of the intervention (ie, the year the voluntary Global Code of Practice on the International Recruitment of Health Personnel [WHO Code] was unanimously signed). B, Mean physician density (per 1000 population) was plotted by countries’ status of physician shortage designation from 2000 to 2021. The dashed line represents the year of the intervention (ie, the year the voluntary WHO Code was unanimously signed).

The study sample included 135 888 physicians from shortage (treatment) countries and 516 030 physicians from nonshortage (control) countries who immigrated to an OECD country from 2000 to 2021 (eTable 3 in Supplement 1). In 2000, there were 3280 physicians from shortage countries who immigrated to an OECD country and 8553 physicians from nonshortage countries who immigrated to an OECD country (Figure 1A). Trends between shortage and nonshortage countries were similar until 2010. From 2010 to 2014, the total number of physicians emigrating from shortage countries decreased considerably from 6142 to 4474, whereas the number of physicians emigrating from nonshortage countries increased slightly from 25 390 to 25 935. After 2014, both groups experienced upward trends in physician emigration. During the same period, physician density was substantially lower in shortage countries than in nonshortage countries, and the gap became larger over the study period (Figure 1B).

**Figure 2.**
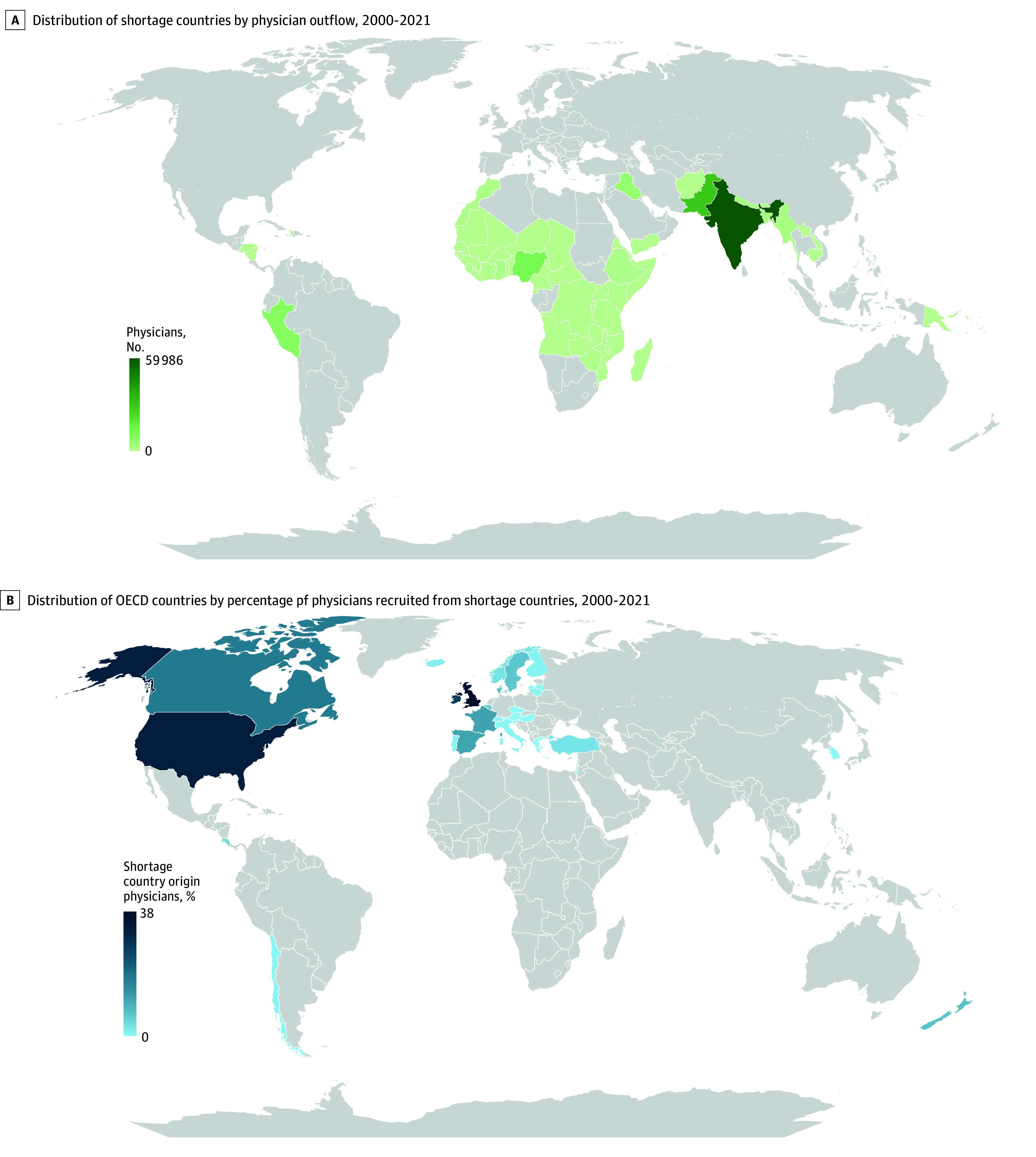
Distribution of the Countries by Physician Outflow and Recruitment From World Health Organization (WHO)–Designated Shortage Countries to Organization for Economic Co-operation and Development (OECD) Countries A, Darker green represents more physicians who moved from WHO-designated shortage countries to OECD countries from 2000 to 2021. Many physicians originated from countries in South Asia and sub-Saharan Africa. B, Darker blue represents more physicians who were recruited from WHO-designated shortage countries to OECD countries from 2000 to 2021. Many physicians immigrated to the UK and the US.

The geographic distribution of physician outflow from treatment (shortage) countries appears in Figure 2A; countries in South Asia, including India (59 986 physicians) and Pakistan (29 852 physicians), had the largest number of physician emigrants during the study period. Other countries with large numbers of physicians emigrating included Nigeria (12 477 physicians), Peru (8934 physicians), and Iraq (5875 physicians). Among the OECD destination countries, the UK had the highest proportion (38%) of physician immigrants from a treatment (shortage) country (Figure 2B). Other destination countries with a sizeable proportion of physicians from treatment countries included the US (33%), Ireland (26%), Canada (17%), France (12%), and Spain (12%).

In the difference-in-differences analysis, there was a decrease in physician outflow by 47.03 physicians (95% CI, −92.29 to −1.76 physicians) per country per year after 2010 in WHO-designated shortage countries compared with nonshortage countries, and the WHO Code was associated with a reduction of nearly 30% in the average annual outflow of physicians from these countries. When the period after introduction of the WHO Code was split, there was a decrease in physician outflow by 59.91 physicians (95% CI, −106.63 to −13.19 physicians) per country per year in the shortage countries from 2010 to 2014 compared with the nonshortage countries but not from 2015 to 2021 (Table 1). When 2015, 2016, or 2017 was used as a cutoff to define the 2 stages after implementation of the WHO Code, the findings were similar; the WHO Code was associated with a reduction during the first stage but not during the second stage (eTable 5 in Supplement 1).

**Table 1.** Difference-in-Differences Regression Estimates of Annual Outflow of Physicians From Before and After the 2010 World Health Organization (WHO) Global Code of Practice on the International Recruitment of Health Personnel (WHO Code) by Physician Shortage Status for the WHO-Designated Shortage and Nonshortage Countries

Outflow of physicians	Shortage countries, estimate			Nonshortage countries, estimate			Difference-in-differences regression estimate (95% CI)	*P* value
	Before WHO Code	After WHO Code	Change	Before WHO Code	After WHO Code	Change		
One single period after introduction of WHO Code from 2010-2021								
Unadjusted model^a^	128.63	128.03	−0.60	128.63	195.91	67.28	−67.88 (−127.81 to −7.94)	.03
Adjusted model^b^	138.57	147.96	9.39	138.57	194.98	56.41	−47.03 (−92.29 to −1.76)	.04
Unadjusted model with 2 distinct periods after introduction of WHO Code^a^								
2010-2014	108.69	90.74	−17.95	137.05	196.35	119.68	−59.30 (−135.28 to −19.22)	.009
2015-2021	108.69	120.49	11.80	137.05	210.03	72.98	−61.18 (−127.89 to 5.53)	.07
Adjusted model with 2 distinct periods after introduction of WHO Code^b^								
2010-2014	135.76	127.75	−8.01	135.76	187.66	51.90	−59.91 (−106.63 to −13.19)	.01
2015-2021	135.76	171.49	35.73	135.76	201.98	66.22	−30.48 (−84.87 to 23.90)	.27

^a^ The models included fixed effects for country and year and the SEs were clustered at the country level.

^b^ Adjusted for male and female mortality per 1000 population, gross national income per capita, foreign direct investment as a percentage of gross domestic product, male and female unemployment rates, health expenditure per capita, out-of-pocket health care expenses per capita, population, and refugee population.

There was a slight decrease in physician density in the WHO-designated shortage countries after 2010 (−0.22 [95% CI, −0.33 to −0.11] physicians per 1000 population) compared with the nonshortage countries (Table 2). In the 2-stage model, there was a reduction in physician density only during the second stage (ie, between 2015 and 2021) (−0.36 [95% CI, −0.51 to −0.22] per 1000 population). The reduction in physician density was only observed during the second stage when 2015, 2016, or 2017 was used as an alternative cutoff to define the 2 stages after 2010 (eTable 5 in Supplement 1).

**Table 2.** Difference-in-Differences Regression Estimates of Physician Density From Before and After the 2010 World Health Organization (WHO) Global Code of Practice on the International Recruitment of Health Personnel (WHO Code) by Physician Shortage Status for the WHO-Designated Shortage and Nonshortage Countries

Physician density	Shortage countries, estimate			Nonshortage countries, estimate			Difference-in-differences regression estimate (95% CI)	*P* value
	Before WHO Code	After WHO Code	Change	Before WHO Code	After WHO Code	Change		
Period after introduction of WHO Code from 2010-2021								
Unadjusted model^a^	1.18	1.93	0.74	1.54	2.65	1.11	−0.37 (−0.47 to −0.28)	<.001
Adjusted model^b^	0.82	1.62	0.80	1.63	2.65	1.02	−0.22 (−0.33 to −0.11)	<.001
Unadjusted model with 2 distinct periods after introduction of WHO Code^a^								
2010-2014	1.32	1.58	0.26	1.69	2.15	0.46	−0.2 (−0.27 to −0.13)	<.001
2015-2021	1.32	1.98	0.66	1.69	2.84	1.15	−0.49 (−0.61 to −0.37)	<.001
Adjusted model with 2 distinct periods after introduction of WHO Code^b^								
2010-2014	0.97	1.27	0.30	1.78	2.16	0.38	−0.08 (−0.17 to 0.013)	.10
2015-2021	0.97	1.68	0.71	1.78	2.85	1.07	−0.36 (−0.51 to −0.22)	<.001

^a^ The models included fixed effects for country and year and the SEs were clustered at the country level.

^b^ Adjusted for male and female mortality per 1000 population, gross national income per capita, foreign direct investment as a percentage of gross domestic product, male and female unemployment rates, health expenditure per capita, out-of-pocket health care expenses per capita, population, and refugee population.

Our findings were similar in the sensitivity analyses that included a study period restriction to 2019, a control group restricted to only LMICs, a 2010 washout period, and in the matched difference-in-differences analysis.

## Discussion

Global health care workforce shortages have been a barrier to achieving universal health coverage. This study evaluated whether the WHO Code achieved its 2010 objective to reduce physician recruitment from WHO-designated shortage countries and to improve the domestic supply of physician in those countries. We found that the introduction of the WHO Code successfully reduced physician outflow from WHO-designated shortage countries to OECD countries in the short-term. On average, the WHO Code was associated with approximately 2600 fewer physicians leaving shortage countries each year or roughly 17 000 physicians during the first 5 years, which is an annual reduction of nearly 30% in physician emigration.

Despite finding a reduction in physician emigration from shortage countries after the WHO Code, we also found that it did not improve the domestic physician supply as measured by physician density (per 1000 population). Our results indicate that even though the voluntary WHO Code may help reduce the absolute quantity of physician recruitment by HICs from shortage countries (thereby achieving the first objective of the WHO Code), further investment is necessary to promote the second objective of supporting physician workforce growth in shortage countries.

Our finding of reduced physician outflow from shortage countries after the WHO Code compared with nonshortage countries may be due to several reasons. There was a substantial push to prioritize the first part of the WHO Code (ie, reducing reliance on LMIC physicians) within the OECD in 2013 by mandating that OECD countries identify alternative solutions to address their domestic physician shortages rather than relying on immigration.^21^ Several countries have identified specific agencies responsible for implementing the WHO Code.^22^ In addition, countries (such as Germany and Norway) took steps to codify parts of the WHO Code into formal policy through bilateral agreements with shortage countries meant to pivot away from active recruitment and pivot toward collaboration that would support domestic workforce development.^9,23^

Despite the reduction in physician outflow, we found that some OECD countries (eg, the UK) may have been less successful in adhering to the WHO Code because they still had large proportions of physicians immigrating from shortage countries throughout our study period. Certain countries (such as India, Nigeria, and Pakistan) have had sizeable physician outflow despite their workforce shortages. This could be due to medical schools within those countries that specifically design their curricula so that their graduates are able to take international physician licensing examinations and emigrate to a HIC.^24^ As previous studies have highlighted, there is a delicate balance between allowing for free migration and combating physician shortages.^25^ Push and pull factors (such as job opportunity and domestic political unrest) have been previously described as reasons why physicians may choose to emigrate.^26^ The WHO acknowledged in its 2010 Code the freedom to migrate for personal and family opportunities while recommending that signatory countries cease active recruitment of physicians from shortage countries. This would allow a pathway for passive autonomous movement of physicians but would limit HICs from explicitly poaching talented physicians from shortage countries. Our findings suggest that this recommendation did reduce physician recruitment from shortage countries by OECD countries, especially in the short run.

Despite the initial reduction in physician outflow from shortage countries, the associations with the WHO Code were attenuated over time. These findings are consistent with a WHO Commission report in 2020 that affirmed the ongoing relevance and importance of the 2010 WHO Code while observing that progress in reducing recruitment has now plateaued, potentially attributable to behavioral changes, loss of passive enforcement, or deprioritization since it was first ratified.^22^ Our findings point to a return to the trends in physician recruitment from shortage countries during the second assessment period after introduction of the WHO Code (from 2015 to 2021). It may be time for HICs to recommit to reducing their reliance on physicians from those countries.

We found declines in physician density (per 1000 population) among the shortage countries after 2010, indicating that the WHO Code was less successful in achieving its second objective of creating a more sustainable physician workforce in the shortage countries. There are multiple contributing factors that likely led to this observed decrease. First, the populations grew faster in shortage countries over our study period than in nonshortage countries.^27^ This would lead to a relative reduction in physician density in shortage countries.

Second, the finding that physician outflow from shortage countries returned to levels before the 2010 introduction of the WHO Code during the second assessment period (from 2015 to 2021) might be a factor contributing to the significant reduction in physician density rates. Furthermore, improving physician density in shortage countries requires longitudinal and resource-heavy investments in those countries. The WHO can provide technical support to shortage countries and promote partnerships among HICs, LMICs, and international organizations. For example, the WHO can leverage existing collaborations such as the Helping Babies Breath program, which is a multinational development program between HICs and LMICs to address neonatal morbidity and mortality.^28^ There are also collaborations between LMICs such as Cuban leasing of their physicians to other LMICs to boost their local workforce because the country is locked out of emigration flow to the US.^29^ The WHO can also coordinate with the World Bank to support physician workforce growth in shortage countries through microfinancing and no interest or low-interest loans. However, shortage countries often appear in the World Bank’s list of fragile and conflict-affected countries, indicating both greater health needs and political instability.^30^ Further study should focus on how conflicts affect physician migration patterns.^31^

Broadly, our study supports the use of the WHO Code as a global health policy lever for reducing physician recruitment by HICs from LMICs and promoting global health equity. Our findings are consistent with prior studies that demonstrated the important role played by the WHO in promoting global health development, especially by supporting LMICs in combating infectious diseases.^32^ Withdrawal from the WHO by the US or other countries may have negative effects on global health development.^33^ Rather than withdrawing from the WHO, countries should take steps to recommit themselves to and bolster their support of this global health governance body, including its efforts to combat physician shortages in LMICs.

The WHO Code established norms for signatory countries in 2010 to commit to reducing their reliance on physician immigrants from shortage countries and supporting physician supply in those countries. In this analysis, we found mixed success of the policy with reduced physician outflow from shortage countries to OECD countries, especially during the first 5 years, but little improvement in domestic physician supply in shortage countries. Our findings point to the important role played by the WHO in promoting global health workforce equity. HICs should recommit to reducing active physician recruitment from shortage countries. Withdrawal from the WHO would exclude certain countries from participating in this important global health governance structure, which is critical to building consensus and multilateral cooperation in the promotion of global health development.

### Limitations

There are limitations to this study. First, there was a lack of information about physician characteristics at the individual level. Second, the WHO first detailed its list of 57 shortage countries in 2006^20^; however, 10 countries were removed from the updated critical shortage list in 2020,^34^ including some of the largest origin countries of physician immigrants (such as India and Pakistan). In subsequent years, the WHO further updated its list of shortage countries, which included some of the previously removed countries. The current study only used countries from the 2006 shortage designation, which could affect long-term evaluation of physician supply in countries that have recently been added to or removed from the shortage list. To assess the potential effect of the WHO list change, a sensitivity analysis was conducted with the study period restricted to before 2020; the results of that analysis were similar to the main results.

Third, the data were limited to physicians immigrating to OECD countries and did not include physicians immigrating to non-OECD countries. In addition, the data did not distinguish movement from one LMIC to another. Fourth, physicians were the focus of this study, and notably, the WHO Code was intended to apply to all health care workers. Future research should examine the associations of the WHO Code with the number of nurses and other types of health professionals.

Fifth, the analysis was unable to capture changes in how health workers are actively recruited over time. Studies suggest recruiters continue to use direct, digital marketing strategies to actively recruit physicians from shortage countries,^35^ contradicting the intended purpose of the WHO Code. Sixth, additional studies should evaluate restrictive immigration laws in HICs and their associations with global migration from LMICs.

## Conclusions

This study found that voluntary implementation of the WHO Code was associated with lower physician outflow from WHO-designated shortage countries without improvement in physician density in those countries.

## Notes

Supplement 1.
eLists
**eTable 1.** Placebo Test Outcomes for Adjusted Annual Outflow of Physicians with A Single Stage in the Post-Period **eTable 2.** Placebo Test Outcomes for Adjusted Physician Density (per 1,000) with A Single Stage in the Post-Period **eTable 3.** Results from the Models with the Sample Restricted to Before 2020 **eTable 4.** Results from the Models with the Control Group Restricted to Low- and Middle-Income Countries **eTable 5.** Results from the Models Using Different Cutoffs to Define Two Stages in the Post-Period **eTable 6.** Results from the Models Using 2010 as the Washout Period **eTable 7.** Results from the Models using a Matched Difference-in-Differences Design

Supplement 2.
Data sharing statement
