# Supplementary material for: Changes in Physician Emigration and Density After the 2010 WHO Global Code of Practice
Source: JAMA Health Forum. 2026 Feb 6;7(2):e256718. doi: 10.1001/jamahealthforum.2025.6718 (PMC12881981; doi:10.1001/jamahealthforum.2025.6718)
Supplement: Supplement 1. — eLists eTable 1. Placebo Test Outcomes for Adjusted Annual Outflow of Physicians with A Single Stage in the Post-Period eTable 2. Placebo Test Outcomes for Adjusted Physician Density (per 1,000) with A Single Stage in the Post-Period eTable 3. Results from the Models with the Sample Restricted to Before 2020 eTable 4. Results from the Models with the Control Group Restricted to Low- and Middle-Income Countries eTable 5. Results from the Models Using Different Cutoffs to Define Two Stages in the Post-Period eTable 6. Results from the Models Using 2010 as the Washout Period eTable 7. Results from the Models using a Matched Difference-in-Differences Design [file jamahealthforum-e256718-s001.pdf]

Supplemental Online Content

Ramesh T, Mukherjee JS, Zhang F, Jena AB, Yu H. Changes in physician emigration and density after the 2010 WHO global code of practice. *JAMA Health Forum*. Published online February 6, 2026. doi:10.1001/jamahealthforum.2025.6718

eLists

eTable 1. Placebo Test Outcomes for Adjusted Annual Outflow of Physicians with A Single Stage in the Post-Period
eTable 2. Placebo Test Outcomes for Adjusted Physician Density (per 1,000) with A Single Stage in the Post-Period
eTable 3. Results from the Models with the Sample Restricted to Before 2020
eTable 4. Results from the Models with the Control Group Restricted to Low- and Middle-Income Countries
eTable 5. Results from the Models Using Different Cutoffs to Define Two Stages in the Post-Period
eTable 6. Results from the Models Using 2010 as the Washout Period
eTable 7. Results from the Models using a Matched Difference-in-Differences Design

This supplemental material has been provided by the authors to give readers additional information about their work.

© 2026 Ramesh T et al. *JAMA Health Forum*.

eLists

Shortage countries: Afghanistan, Angola, Bangladesh, Benin, Bhutan, Burkina Faso, Burundi, Cambodia, Cameroon, Central African Republic, Chad, Comoros, Congo, Côte d'Ivoire, Democratic Republic of the Congo, Djibouti, El Salvador, Equatorial Guinea, Eritrea, Ethiopia, Gambia, Ghana, Guinea, Guinea-Bissau, Haiti, Honduras, India, Indonesia, Iraq, Kenya, Laos, Lesotho, Liberia, Madagascar, Malawi, Mali, Mauritania, Morocco, Mozambique, Myanmar, Nepal, Nicaragua, Niger, Nigeria, Pakistan, Papua New Guinea, Peru, Rwanda, Senegal, Sierra Leone, Somalia, Tanzania, Togo, Uganda, Yemen, Zambia, and Zimbabwe

Nonshortage countries: Algeria, Andorra, Antigua and Barbuda, Argentina, Armenia, Australia, Austria, Azerbaijan, Bahamas, Bahrain, Barbados, Belarus, Belgium, Belize, Bolivia, Bosnia and Herzegovina, Botswana, Brazil, Brunei, Bulgaria, Cabo Verde, Canada, Chile, China, Colombia, Costa Rica, Croatia, Cuba, Cyprus, Czechia, Denmark, Dominica, Dominican Republic, Ecuador, Egypt, Estonia, Fiji, Finland, France, Gabon, Georgia, Germany, Greece, Grenada, Guatemala, Guyana, Hungary, Iceland, Iran, Ireland, Israel, Italy, Jamaica, Japan, Jordan, Kazakhstan, Kiribati, Kuwait, Kyrgyzstan, Latvia, Lebanon, Libya, Lithuania, Luxembourg, Malaysia, Maldives, Malta, Marshall Islands, Mauritius, Mexico, Micronesia, Moldova, Monaco, Mongolia, Montenegro, Namibia, Nauru, Netherlands, New Zealand, North Macedonia, Norway, Oman, Palau, Panama, Paraguay, Philippines, Poland, Portugal, Qatar, Romania, Russia, Saint Kitts and Nevis, Saint Lucia, Saint Vincent and the Grenadines, Samoa, San Marino, Sao Tome and Principe, Saudi Arabia, Serbia, Seychelles, Singapore, Slovakia, Slovenia, Solomon Islands, South Africa, South Korea, South Sudan, Spain, Sri Lanka, Sudan, Suriname, Swaziland, Sweden, Switzerland, Syria, Tajikistan, Thailand, Timor-Leste, Tonga, Trinidad and Tobago, Tunisia, Turkey, Turkmenistan, Tuvalu, Ukraine, United Arab Emirates, UK, US, Uruguay, Uzbekistan, Vanuatu, Venezuela, and Vietnam

© 2026 Ramesh T et al. *JAMA Health Forum*.

eTable 1. Placebo Test Outcomes for Adjusted Annual Outflow of Physicians with A Single Stage in the Post-Period

	ATET	p value	95% Confidence Interval	
2001	-22.22	0.57	-98.39	53.95
2002	-21.02	0.67	-118.89	76.85
2003	-30.17	0.47	-112.27	51.93
2004	-15.14	0.64	-79.81	49.53
2005	-37.08	0.22	-96.13	21.97
2006	-51.75	0.20	-131.14	27.65
2007	-53.20	0.18	-130.78	24.37
2008	-49.13	0.17	-119.51	21.25

© 2026 Ramesh T et al. *JAMA Health Forum*.

eTable 2. Placebo Test Outcomes for Adjusted Physician Density (per 1,000) with A Single Stage in the Post-Period

	**ATET**	**p value**	**95% Confidence Interval**	
2001	-20.47	0.61	-100.43	59.50
2002	-27.01	0.41	-91.77	37.76
2003	-15.48	0.55	-65.99	35.03
2004	-31.08	0.15	-73.49	11.33
2005	-42.78	0.12	-96.04	10.48
2006	-46.58	0.09	-99.81	6.65
2007	-48.00	0.07	-100.31	4.32
2008	-48.00	0.07	-100.31	4.32

© 2026 Ramesh T et al. *JAMA Health Forum*.

eTable 3. Results from the Models with the Sample Restricted to Before 2020

Adjusted Models	ATET	p value	95% Confidence Interval	
A Model with A Single Stage in the Post-Period Annual Outflow of Physicians	-46.05	0.051	-92.37	0.27
A Model with Two Stages in the Post-Period Annual Outflow of Physicians (2010-2014)	-56.24	0.019	-103.20	-9.28
A Model with Two Stages in the Post-Period Annual Outflow of Physicians (2015-2019)	-26.80	0.369	-85.55	31.96
A Model with A Single Stage in the Post-Period Physician Density (per 1,000)	-0.20	< 0.001	-0.31	-0.10
A Model with Two Stages in the Post-Period Physician Density (per 1,000); 2010-2014	-0.11	0.012	-0.20	-0.03
A Model with Two Stages in the Post-Period Physician Density (per 1,000); 2015-2019	-0.35	< 0.001	-0.50	-0.20

In this sensitivity analysis, we used a study period restriction to before 2020 for our adjusted models.

© 2026 Ramesh T et al. *JAMA Health Forum*.

eTable 4. Results from the Models with the Control Group Restricted to Low- and Middle-Income Countries

**Adjusted Models**	**ATET**	**p value**	**95% Confidence Interval**	
A Model with A Single Stage in the Post-Period Annual Outflow of Physicians	-43.71	0.063	-89.82	2.39
A Model with Two Stages in the Post-Period Annual Outflow of Physicians (2010-2014)	-46.25	0.032	-88.37	-4.14
A Model with Two Stages in the Post-Period Annual Outflow of Physicians (2015-2021)	-40.42	0.244	-108.74	27.90
A Model with A Single Stage in the Post-Period Physician Density (per 1,000)	-0.15	0.047	-0.30	0.00
A Model with Two Stages in the Post-Period Physician Density (per 1,000); 2010-2014	-0.07	0.313	-0.19	0.06
A Model with Two Stages in the Post-Period Physician Density (per 1,000); 2015-2021	-0.26	0.0140	-0.48	-0.05

© 2026 Ramesh T et al. *JAMA Health Forum*.

eTable 5. Results from the Models Using Different Cutoffs to Define Two Stages in the Post-Period

Changing Split Period	ATET	p value	95% Confidence Interval	
2015 as the Cutoff				
Annual Outflow of Physicians (2010-2015)	-61.81	0.013	-110.58	-13.04
Annual Outflow of Physicians (2016-2021)	-31.01	0.312	-91.38	29.35
2016 as the Cutoff				
Annual Outflow of Physicians (2010-2016)	-59.84	0.016	-108.40	-11.28
Annual Outflow of Physicians (2017-2021)	-27.48	0.405	-92.48	37.52
2017 as the Cutoff				
Annual Outflow of Physicians (2010-2017)	-56.64	0.021	-104.45	-8.84
Annual Outflow of Physicians (2018-2021)	-26.78	0.459	-97.95	44.39
2015 as the Cutoff				
Physician Density (per 1,000); 2010-2015	-0.10	0.033	-0.19	-0.01
Physician Density (per 1,000); 2016-2021	-0.39	< 0.001	-0.54	-0.23
2016 as the Cutoff				
Physician Density (per 1,000); 2010-2016	-0.12	0.009	-0.21	-0.03
Physician Density (per 1,000); 2017-2021	-0.42	< 0.001	-0.59	-0.25
2017 as the Cutoff				
Physician Density (per 1,000); 2010-2017	-0.15	0.003	-0.24	-0.05
Physician Density (per 1,000); 2018-2021	-0.45	< 0.001	-0.62	-0.27

© 2026 Ramesh T et al. *JAMA Health Forum*.

eTable 6. Results from the Models Using 2010 as the Washout Period

	**ATET**	**p value**	**95% Confidence Interval**	
An Unadjusted Model with A Single Stage in the Post-Period Annual Outflow of Physicians	-69.32	0.033	-132.83	-5.82
An Adjusted Model with A Single Stage in the Post-Period Annual Outflow of Physicians	-48.07	0.056	-97.48	1.33
An Adjusted Model with Two Stages in the Post-Period Annual Outflow of Physicians (2010-2014)	-69.20	0.011	-122.13	-16.28
An Adjusted Model with Two Stages in the Post-Period Annual Outflow of Physicians (2015-2021)	-32.92	0.258	-90.19	24.35
An Unadjusted Model with A Single Stage in the Post-Period Physician Density (per 1,000)	-0.39	< 0.001	-0.49	-0.29
An Adjusted Model with A Single Stage in the Post-Period Physician Density (per 1,000)	-0.25	< 0.001	-0.37	-0.13
An Adjusted Model with Two Stages in the Post-Period Physician Density (per 1,000); 2010-2014	-0.08	0.133	-0.18	0.02
An Adjusted Model with Two Stages in the Post-Period Physician Density (per 1,000); 2015-2021	-0.37	< 0.001	-0.51	-0.22

© 2026 Ramesh T et al. *JAMA Health Forum*.

eTable 7. Results from the Models using a Matched Difference-in-Differences Design

	ATET	p value	95% Confidence Interval	
A Model with A Single Stage in the Post-Period<br>Annual Outflow of Physicians	-72.44	0.035	-139.66	-5.23
A Model with Two Stages in the Post-Period<br>Annual Outflow of Physicians (2010-2014)	-85.99	0.013	-153.60	-18.37
A Model with Two Stages in the Post-Period<br>Annual Outflow of Physicians (2015-2021)	-62.24	0.095	-135.47	11.00
A Model with A Single Stage in the Post-Period<br>Physician Density (per 1,000)	-0.37	< 0.001	-0.47	-0.27
A Model with Two Stages in the Post-Period<br>Physician Density (per 1,000); 2010-2014	-0.19	< 0.001	-0.27	-0.11
A Model with Two Stages in the Post-Period<br>Physician Density (per 1,000); 2015-2021	-0.50	< 0.001	-0.63	-0.37

With nearest neighbor matching with replacement on our covariates (i.e. male and female mortality per 1,000, GNI per capita, foreign direct investment, male and female unemployment rates, health expenditure per capita, out of pocket healthcare expenses per capita, population, and refugee population.

© 2026 Ramesh T et al. *JAMA Health Forum*.
