# Supplementary material for: Changes in Physician Emigration and Density After the 2010 WHO Global Code of Practice
Source: JAMA Health Forum. 2026 Feb 6;7(2):e256718. doi: 10.1001/jamahealthforum.2025.6718 (PMC12881981; doi:10.1001/jamahealthforum.2025.6718)
Supplement: Supplement 2. — Data sharing statement [file jamahealthforum-e256718-s002.pdf]

Data Sharing Statement

Ramesh. Changes in Physician Emigration and Density After the 2010 WHO Global Code of Practice. *JAMA Health Forum*. Published February 06, 2026.
doi:10.1001/jamahealthforum.2025.6718

Data
Data available: Yes
Data types: Data (not involving human participants)
How to access data: https://data-explorer.oecd.org/
When available: With publication

Supporting Documents
Document types: Statistical/analytic code
How to access documents: https://data-explorer.oecd.org/
When available: With publication

Additional Information
Who can access the data: researchers whose proposed use of the data has been approved
Types of analyses: researchers whose proposed use of the data has been approved
Mechanisms of data availability: researchers whose proposed use of the data has been approved
